# Comparative effectiveness of six Chinese herb formulas for acute exacerbation of chronic obstructive pulmonary disease: a systematic review and network meta-analysis

**DOI:** 10.1186/s12906-019-2633-2

**Published:** 2019-08-22

**Authors:** Shaonan Liu, Jing Chen, Jinhong Zuo, Jiaqi Lai, Lei Wu, Xinfeng Guo

**Affiliations:** 1 The Second Affiliated Hospital of Guangzhou University of Chinese Medicine, Guangzhou, China; 2 The Second Clinical College of Guangzhou University of Chinese Medicine, Guangzhou, China; 3 Guangdong Provincial Hospital of Chinese Medicine, Guangzhou, China; 4 Guangdong Provincial Academy of Chinese Medical Sciences, No.111, Dade Road, Guangzhou, China; 5 Zhongshan City People’s Hospital, Zhongshan, China

**Keywords:** Chinese herb formula, AECOPD, Network meta-analysis

## Abstract

**Background:**

Six Chinese herb formulas, namely, the Weijing decoction (WJ), the Maxingshigan decoction (MXSG), the Yuebijiabanxia decoction (YBBX), the Qingqihuatan decoction (QQHT), the Dingchuan decoction (DC) and the Sangbaipi decoction (SBP), are commonly used, along with routine pharmacotherapy, to manage the acute exacerbation of chronic obstructive pulmonary disease (AECOPD). In this study, we conducted a systematic review to summarize the efficacy of these six formulas, and we also conducted a network meta-analysis (NMA) to rank these formulas.

**Methods:**

We searched five English databases and four Chinese databases, with dates ranging from the starting dates of these databases to December 2016. Randomized controlled trials that evaluated any of the six Chinese herb formulas combined with the use of pharmacotherapy for AECOPD were identified.

**Results:**

Fifty-five studies involving 4560 participants were included. The pairwise meta-analyses showed that WJ and QQHT had superior effects on the improvement of lung function (forced expiratory volume in 1 seconds; FEV1) (mean difference (MD): 0.25, 95% confidence interval (CI): 0.19–0.30 and 0.34, 95%CI: 0.10–0.58). MXSG, WJ and QQHT were found to be more effective for improving arterial blood gases (PaO2 and PaCO2). In terms of effective rates, all of these formulas had additional favourable effects compared to routine pharmacotherapy. The results of the NMA analyses indicated that only MXSG showed superior add-on effects for the improvement of FEV1 (MD: 0.37, 95% credible interval (CrI): 0.03–0.72). Most of the formulas combined with routine pharmacotherapy were superior to pharmacotherapy alone for the improvement of arterial blood gases and effective rates. The ranking tests suggested that QQHT and MXSG combined with routine pharmacotherapy might be optimal options for the treatment of AECOPD.

**Conclusions:**

This NMA indicated that QQHT and MXSG might be more effective treatment regimens for AECOPD. Further well-designed studies that specifically examine the direct comparisons of these formulas are needed to support our conclusions.

**Electronic supplementary material:**

The online version of this article (10.1186/s12906-019-2633-2) contains supplementary material, which is available to authorized users.

## Background

Chronic obstructive pulmonary disease (COPD) is a worldwide disease with a high mortality and morbidity burden and will become the third leading cause of worldwide death in 2030 [1]. Acute exacerbation of COPD (AECOPD) is defined as an acute worsening of respiratory symptoms, which is associated with the decline of patients’ health statuses and lung function and can even increase the risk of death [2, 3]. Pharmacological interventions that are recommended in the clinical guidelines have exhibited effects on preventing and managing exacerbations. Unfortunately, AECOPD still occurs frequently, and death is a common outcome for hospitalized patients [4]. Thus, further treatment strategies, including integrated Chinese medicine and routine pharmacology (RP), are still urgently needed for AECOPD management.

Chinese herbal medicine has been used to manage respiratory diseases for thousands of years and is commonly prescribed in clinical practice for use in combination with western medicine techniques for patients with AECOPD. Various strategies are recommended in the Chinese clinical guidelines [5–7], including the use of six commonly used Chinese herb formulas: the Weijing decoction (WJ), the Maxingshigan decoction (MXSG), the Yuebijiabanxia decoction (YBBX), the Qingqihuatan decoction (QQHT), the Dingchuan decoction (DC) and the Sangbaipi decoction (SBP). Previous systematic reviews have demonstrated the efficacies of each of the previously mentioned formulas [8, 9]. Additionally, mechanistic studies have revealed the anti-inflammation and antioxidative stress functions of these formulas and the active compounds of the herb ingredients [10–12]. However, it is still unknown which formula is superior when combined with routine pharmacotherapy.

As a new meta-analytic technique, a network meta-analysis can estimate both direct and indirect comparisons and can provide a ranking of optimal interventions [13–15]. Therefore, we conducted this systematic review and a network meta-analysis to provide a ranking of the frequently used Chinese formulas for the management of AECOPD.

## Methods

The protocol of this study was registered on PERSPERO (CRD42016052699) and the full text was published elsewhere [16]. We reported this review by following the Preferred Reporting Items for Systematic Reviews and Meta-Analysis (PRISMA) extension statement for network meta-analyses of health care interventions [17] (Additional file 1).

### Eligibility criteria

We included randomized controlled trials of patients with acute exacerbation of COPD that investigated the efficacies of six Chinese herb formulas combined with routine pharmacotherapy. Patients with AECOPD were confirmed to have the disorder via global standard diagnostic criteria and clinical symptoms [18]. Intervention regimens consisted of pharmacotherapy combined with the following formulas: WJ, MXSG, YBBX, QQHT, DC and SBP. Studies were included in the analysis if they reported one or more of the following pre-defined outcomes. The primary outcomes were FEV1, PaO2, PaCO2 and length of hospital stay. The secondary outcomes included (1) dyspnoea; (2) health-related quality of life; (3) hospital readmission for acute exacerbation; (4) effective rate [19]; and (5) adverse events. Studies were excluded if (1) a study included participants with complications of COPD, such as cor pulmonale or pulmonary hypertension or (2) data were unavailable.

### Search strategy

We searched PubMed, EMBASE, the Cochrane Central Register of Controlled Trials (CENTRAL), CINAHL, AMED, the Chinese Biomedical Database (CBM), the Chinese National Knowledge Infrastructure (CNKI), the Chongqing VIP information (CQVIP) and the Wanfang database from the starting dates of these databases to December 2016. Furthermore, the reference lists of the retrieved systematic reviews and the included studies were also screened. Language restrictions were not applied. The search terms are presented in Additional file 2.

### Study selection and data extraction

Two independent reviewers (JL and JZ) screened the titles and abstracts of the citations and evaluated the full texts according to the selection criteria. Any disagreement was resolved by discussion with a third reviewer (JC).

Data extraction was performed by using the Epidata software 3.1 with a pre-designed database sheet. The following variables were included for the analysis: first author, publication year, diagnosis information, disease duration, stage, sample size, age, details of the interventions, control, outcomes, treatment duration, follow-up period and adverse events.

### Risk of bias assessment

Two researchers (JL and JZ) independently evaluated the biases of the eligible studies by using the Cochrane collaboration of risk of bias tool. Six items (including randomization methods, allocation concealment, the blinding of participants and personnel, the blinding of the outcome assessors, incomplete outcome data, selective reporting and other sources of bias) were assessed, and any discrepancies were resolved by consensus.

### Statistical analysis

We conducted a traditional, pair-wise meta-analysis using a random-effects model. For the continuous data, such as FEV1, mean differences were estimated, along with 95% confidence intervals (CIs), for the individual studies. Effective rates, as the dichotomous data, were reported with odds ratios (ORs) and 95%CIs. The chi-squared test and I^2^ test were used to assess the heterogeneity.

We conducted the NMA by using the Markov Chain Monte Carlo methods to compare the treatment effects of the six Chinese herb formulas. The posterior distribution was calculated by using 100,000 iterations, with a first burn-in of 20,000 iterations. The consistency between the direct and indirect comparisons was not determined because direct evidence was unavailable. The adjusted indirect comparisons were estimated with a 95% credible interval (95%CrI) for the dichotomous and continuous outcomes. To investigate the best interventions for the various treatments, a surface under the cumulative ranking curve (SUCRA) and mean ranks were performed for the main outcomes. We also generated a comparison-adjusted funnel plot to investigate the potential publication bias. The network meta-analysis was conducted by using the WinBUGS 1.4.3 software, and the main relative graphs were created by using Stata 13.1 software.

## Results

### Literature search and basic characteristics

**Fig. 1:**
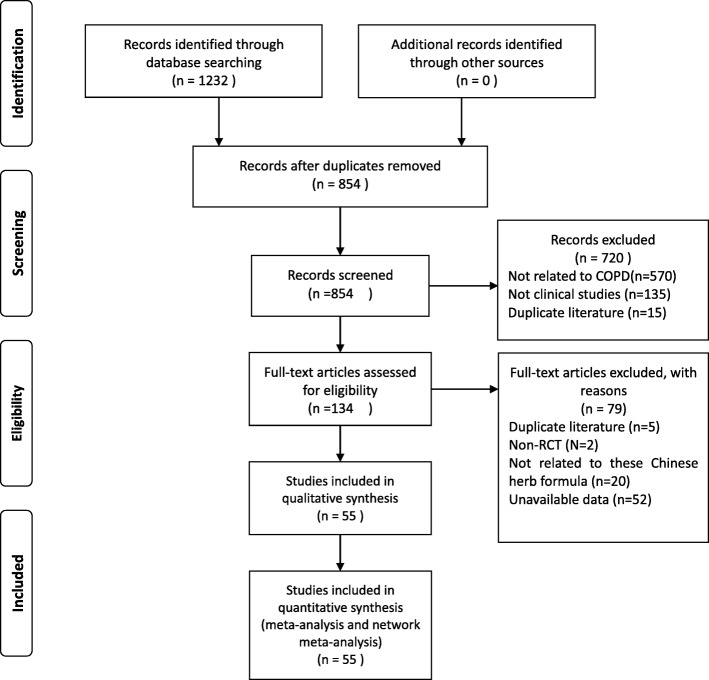
Flowchart of searching and screening for the studies

The literature search identified a total of 1232 citations, and 55 studies involving 4560 participants met the eligible criteria and were included in the analyses (Fig. 1) [20–74]. All of the included studies diagnosed the participants according to the Chinese COPD guidelines. Twenty-six of the included studies reported the severity of the condition at baseline, ranging from mild to very severe. The mean age of the patients with AECOPD was 63.76 years. The mean duration of the condition ranged from 173.47 days [64] to 22.65 years [67].

For the intervention of the Chinese herb formulas, MXSG was the most frequently reported treatment (15 trials), followed by WJ (12 trials), SBP (10 trials), QQHT (9 trials), DC (6 trials) and YBBX (3 trials). The formula ingredients and scientific names of herbs are listed in Additional file 3. In clinical practice, a formula is usually modified based on the original/classical formula according to the theory of Chinese medicine and a patient’s clinical symptoms. The herb details of included studies are presented in Additional file 4. The treatment duration ranged from 5 days [70] to 30 days [20]. Only one study reported the follow-up period and investigated the recurrence after 1 month of treatment [41].

FEV1 as the primary outcome was reported in 18 studies. The most frequently investigated outcome was the effective rate, which was evaluated in 52 studies. Other outcomes, such as hospital admissions and lengths of hospital stay, were not reported in any of the included studies. The additional characteristics of the included studies are summarized in Table 1.

**Table 1:** Characteristics of included studies

Study ID	Age(year) (Mean ± SD)	Severity	Duration of condition(year) ((Mean ± SD))	Sample size randomised/assessed	intervention	control	Treatment duration	outcomes	Adverse events
Zou HD 2015 [20]	I:65.32 ± 15.41; C:68.40 ± 16.22	1–4	I:14.12 ± 5.14; C:15.24 ± 3.05	I:50/50; C:50/50	MXSG+RP	RP	30d	FEV1;PaO2;PaCO2;ER	0
Zhou ZJ 2016 [21]	I:52.8 ± 4.7; C:52.7 ± 4.9	NR	NR	I:40/40; C:40/40	MXSG+RP	RP	7d	ER	NR
Zhou YH 2015 [22]	I:67.73 ± 15.04; C:64.30 ± 14.19	NR	I:9.52 ± 3.33; C:10.8 ± 3.78	I:44/44; C:40/40	DC + RP	RP	3w	FEV1;ER;mMRC	0
Zhou KL 2016 [23]	NR	NR	NR	I:60/60;C:60/60	MXSG+RP	RP	10d	ER;FEV1%	NR
Zheng XM 2014 [24]	I:71.2 ± 8.01; C:72.30 ± 8.98	2–3	I:4~40; C:3~30	I:30/30; C:30/30	SBP + RP	RP	10d	FEV1%;PaO2;PaCO2;ER	1
Zhao WH 2007 [25]	I:67.37 ± 6.03; C:67.27 ± 5.75	1–3	I: 3.62 ± 2.07; C: 3.13 ± 1.99	I:30/30;C:30/30 mMRC	WJ + RP	RP	10d	ER;PaO2;PaCO2	0
Zhang LS 2011 [26]	67.7 ± 7.1	NR	NR	I:40/40;C:40/40	WJ + RP	RP	10d	FEV1;FEV1%;6MWD	18
Zhang JH 2012 [27]	I:64.21 ± 6.12; C:64.30 ± 6.13	1–2	I:10.01 ± 0.89; C:10.05 ± 0.32	I:30/30;C:30/30	QQHT+RP	RP	10d	ER;FEV1;FEV1%	0
Zhang JH 2006 [29]	I:65.21 ± 6.02; C:65.30 ± 6.13	1–2	NR	I:30/30;C:30/30	WJ + RP	RP	10d	ER;FEV1;FEV1%;PaO2; PaCO2	0
Zhang J 2011 [28]	I:55.4 ± 11.6; C:54.8 ± 8.54	1–2	I:10~30; C:10~30	I:50/50;C:50/50	MXSG+RP	RP	2w	ER;FEV1%, PaO2	0
Zhang CM 2012 [30]	I:55~82; C:53~84	2	I:3~30; C:3~30	I:44/44;C:44/44	SBP + RP	RP	10d	ER	0
Zhang CL 2016 [31]	I:63.1 ± 10.5; C:64.5 ± 8.9	NR	I:15.3 ± 7.2; C:14.81 ± 8.3	I:48/48;C:48/48	YBBX+RP	RP	14d	ER;6MWD	NR
Ye L 2011 [32]	NR	NR	NR	I:30/30;C:30/30	SBP + RP	RP	10d	ER;FEV1%;PaO2;PaCO2	NR
Yang HW 2012 [33]	I:44~78; C:45~79	1–3	I:4~22; C:4~20	I:30/30;C:30/30	MXSG+RP	RP	15d	ER;PaO2;PaCO2	0
Xie WH 2009 [34]	I:45~79; C:44~80	1–3	I:4~20; C:3~19	I:42/42;C:40/40	MXSG+RP	RP	15d	ER;FEV1	NR
Xie JJ 2011 [35]	I:60 ± 3.55; C:61.5 ± 4.38	1–3	I:5~20; C:4~21	I:40/40;C:40/40	YBBX+RP	RP	15d	ER	NR
Wang XP 2015 [36]	I:58.32 ± 15.21; C:52.64 ± 15.74	NR	NR	I:39/39;C:39/39	MXSG+RP	RP	2w	ER;FEV1	NR
Wang PC 2012 [37]	I:45~82; C:47~83	1–3	I:3~28; C:4~25	I:35/35;C:35/35	YBBX+RP	RP	2w	FEV1%	NR
Wang CH 2015 [38]	NR	NR	NR	I:30/30;C:30/30	MXSG+RP	RP	10d	ER	NR
Wang BH 2016 [39]	I:66.58 ± 2.5; C:66.42 ± 2.37	NR	I:8.92 ± 1.41; C:8.88 ± 1.34	I:42/42;C:42/42	WJ + RP	RP	NR	ER;FEV1;FEV1%;PaO2; PaCO2	NR
Sun XS 2015 [40]	I:63.2 ± 9.7; C:61.9 ± 9.1	2–3	I:10.3 ± 4.9; C:9.7 ± 4.6	I:106/106; C:106/106	MXSG+RP	RP	14d	ER;FEV1%	NR
Sun JF 2012 [41]	NR	NR	NR	I:31/31;C:31/31	DC + RP	RP	14d;	ER	NR
Shi YY 2005 [42]	I:61.4 ± 6.8; C:59.5 ± 7.2	1–3	I:16.34 ± 9.53; C:17.17 ± 10.22	I:40/40;C:30/30	WJ + RP	RP	14d	ER;FEV1;FEV1%;PaO2; PaCO2	0
Ma DN 2013 [43]	I:71.74 ± 7.67; C:73.58 ± 5.31	1–4	I:21.13 ± 12.20; C:21.58 ± 10.59	I:40/39;C:40/40	SBP + RP	RP	10d	ER;FEV1;FEV1%;PaO2; PaCO2	NR
Lv T 2014 [44]	I:57.7 ± 2.6; C:57.4 ± 2.3	NR	I:10.5 ± 2.8; C:10.2 ± 2.4	I:104/104;C:104/104	QQHT+RP	RP	14d	ER;FEV1;FEV1%	NR
Liu X 2011 [45]	I:70.03 ± 6.22; C:70.90 ± 5.45	1–3	I:9.03 ± 3.36; C:8.65 ± 2.47	I:30/30;C:30/30	DC + RP	RP	7d	ER;FEV1;FEV1%	0
Liu JB 2006 [46]	I:69.17 ± 7.53; C:69.05 ± 7.83	1–3	I:16.86 ± 10.97; C:17.2 ± 11.25	I:30/30;C:30/30	WJ + RP	RP	10d	ER;FEV1%	NR
Lin YZ 2014 [47]	I:64.1 ± 4.2; C:62.5 ± 3.1	2–3	I:14.1 ± 3.5; C:13.7 ± 3.5	I:102/102;C:90/90	MXSG+RP	RP	7d	ER;FEV1%	NR
Lin J 2011 [48]	I:70.2 ± 5.4; C:68.6 ± 4.2	NR	NR	I:42/42;C:40/40	DC + RP	RP	10d	ER;FEV1;FEV1%; PaO2; PaCO2	NR
Li ZR 2016 [49]	I:67.2; C:65.15	NR	I:6~23; C:7~25	I:20/20;C:20/20	SBP + RP	RP	10d	PaO2; PaCO2	NR
Li YM 2012 [50]	I:61.89 ± 9.67; C:63.71 ± 8.12	1–4	I:16.54 ± 12.65; C:15.93 ± 11.15	I:40/40;C:40/40	SBP + RP	RP	10d	ER;FEV1;FEV1%	0
Li Y 2013 [51]	I:62.17 ± 7.53; C:64.05 ± 7.83	1–3	I:13.86 ± 10.97; C:17.2 ± 11.25	I:30/30;C:30/30	QQHT+RP	RP	10d	ER;PaO2;PaCO2	NR
Li XC 2014 [52]	I:68.11 ± 6.25; C:66.98 ± 6.70	NR	I:14.68 ± 6.32; C:15.95 ± 5.71	I:32/31;C:32/30	SBP + RP	RP	7d	ER;PaO2;PaCO2	0
Li SQ 2013 [53]	I:65.3 ± 8.2; C:64.8 ± 8.6	NR	I:9.6 ± 4.2; C:9.2 ± 4.7	I:40/40;C:40/40	QQHT+RP	RP	10d	ER	0
Li L 2016 [54]	I:62.3 ± 11.2; C:63.1 ± 10.5	NR	I:11.20 ± 10.80; C:11.90 ± 10.90	I:40/40;C:40/40	MXSG+RP	RP	14d	ER	0
Li HM 2012 [55]	I:66.6 ± 6.62; C:65.50 ± 7.16	1–3	I:12.6 ± 4.14; C:13.22 ± 4.28	I:30/30;C:30/30	QQHT+RP	RP	14d	ER;FEV1%	0
Ju P 2015 [56]	I:68.56 ± 6.43; C:70.32 ± 7.82	NR	I:20.27 ± 5.03; C:20.35 ± 5.72	I:30/30;C:30/30	QQHT+RP	RP	14d	ER;PaO2;PaCO2	NR
Jing X 2011 [57]	I:55.63 ± 6.68; C:56.50 ± 5.89	2–3	I:19.79 ± 7.28; C:18.21 ± 9.02	I:24/24;C:24/24	MXSG+RP	RP	2w	ER;FEV1%	0
Jing XL 2009 [58]	I:64.32 ± 5.53; C:63.27 ± 5.18	NR	NR	I:37/37;C:37/37	WJ + RP	RP	10d	ER;PaO2; PaCO2	0
Jing XL 2007 [59]	I:66.3 ± 5.4; C:67.2 ± 4.4	1–2	I:17.2 ± 3.5; C:13.5 ± 3.7	I:30/30;C:30/30	WJ + RP	RP	7d	ER;FEV1;PaO2;PaCO2	0
Jiang H 2015 [60]	I:57.6 ± 10.2; C:58.1 ± 10.1	NR	I:10.5 ± 2.6; C:10.8 ± 2.7	I:60/60;C:60/60	MXSG+RP	RP	10d	ER;FEV1%	NR
Jia JY 2016 [61]	I:60~80; C:57~70	NR	I:15~25; C:13~23	I:32/32;C:32/32	QQHT+RP	RP	14d	ER;FEV1;FEV1%;PaO2; PaCO2	NR
Huang XB 2013 [62]	I:62.00 ± 11.40; C:64.00 ± 12.30	NR	I:14.0 ± 7.10(m); C:15.0 ± 8.70(m)	I:35/35;C:35/35	SBP + RP	RP	10d	ER	0
Hua WS 2017 [63]	61.57 ± 6.54	NR	15.0 ± 3.0	I:40/40;C:40/40	MXSG+RP	RP	14d	ER;FEV1%	NR
Hu J 2015 [64]	I:49.17 ± 12.88; C:43.84 ± 16.47	NR	I:173.47 ± 17.20(d); C:175.7 ± 15.75(d)	I:80/80;C:80/80	MXSG+RP	RP	1 m	ER	NR
Guo YY 2010 [65]	I:63.5; C:68.5	NR	I:8.9;C:9.3	I:56/56;C:44/44	WJ + RP	RP	15d	ER	NR
Guo F 2012 [66]	NR	NR	NR	I:34/34;C:34/34	SBP + RP	RP	10d	ER;PaO2;PaCO2	NR
Gao X 2017 [67]	I:60.34 ± 8.27; C:62.44 ± 10.09	NR	I:22.65 ± 9.18; C:21.27 ± 9.97	I:20/20;C:20/20	DC + RP	RP	4w	ER;FEV1;6MWD	NR
Fan HL 2003 [68]	I:65.87 ± 9.07; C:64.92 ± 8.96	1–3	I:20.55 ± 5.84; C:20.06 ± 5.89	I:57/57;C:50/50	QQHT+RP	RP	10d	ER	NR
Chen XM 2009 [69]	I:45~90; C:47~85	NR	NR	I:31/31;C:31/31	WJ + RP	RP	15d	ER;FEV1%;PaO2;PaCO2	0
Chen XP 2016 [70]	I:69.54 ± 7.79; C:68.56 ± 6.27	NR	I:5~10; C:5~10	I:65/65;C:62/62	SBP + RP	RP	5d	ER	2
Chen HY 2012 [71]	I:72.07 ± 8.39; C:71.05 ± 7.93	2–3	I:16.79 ± 10.53; C:17.2 ± 11.25	I:30/30;C:30/30	WJ + RP	RP	2w	ER;FEV1%;PaO2;PaCO2	NR
Bi WZ 2016 [72]	I:72.32 ± 6.14; C:72.12 ± 6.21	NR	I:7.12 ± 2.14; C:7.26 ± 2.01	I:44/44;C:44/44	DC + RP	RP	14d	ER	0
Jing XL 2006 [73]	I:64.7 ± 5.2; C:64.2 ± 4.9	1–2	I:13.2 ± 3.7; C:12.8 ± 3.9	I:30/30;C:30/30	WJ + RP	RP	10d	ER;FEV1	0
Wang X 2010 [74]	I:47.7 ± 4.0; C:47.2 ± 3.64	1–2	I:11.7 ± 2.85; C:12.6 ± 2.13	I:30/30;C:30/30	QQHT+RP	RP	14d	ER;FEV1%	0

*WJ* Weijing decoction, *SBP* Sangbaipi decoction, *YBBX* Yuebijiabanxia decoction, *DC* Dingchuan decoction, *QQHT* Qingqihuatan decoction, *MXSG* Maxingshigan decoction, *RP* Routine Pharmacotherapy, *NR* not reported, *mMRC* modified medical research council dyspnoea scale, *6MWD* 6-min walk distance, *m* month, *d* day, *w* week

### Risk of bias

**Fig. 2:**
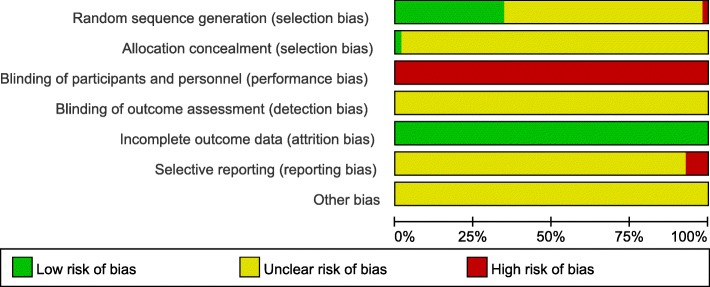
Risk of bias graph

Nineteen studies (35%) reported an appropriate method for randomization, and allocation concealment was only reported in one study. None of the studies made efforts to blind the personnel or the participants. In terms of selective outcome reporting, four studies were assessed as being high risk because several of the pre-specified outcomes were not reported in their results. The risk of bias summary is listed in Fig. 2.

### Treatment efficacy

#### FEV1

**Fig. 3:**
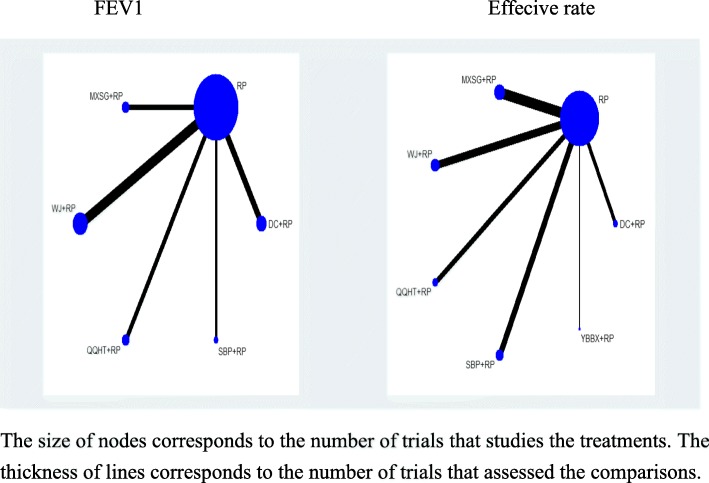
Network diagram for FEV1 and effective rate

**Fig. 4:**
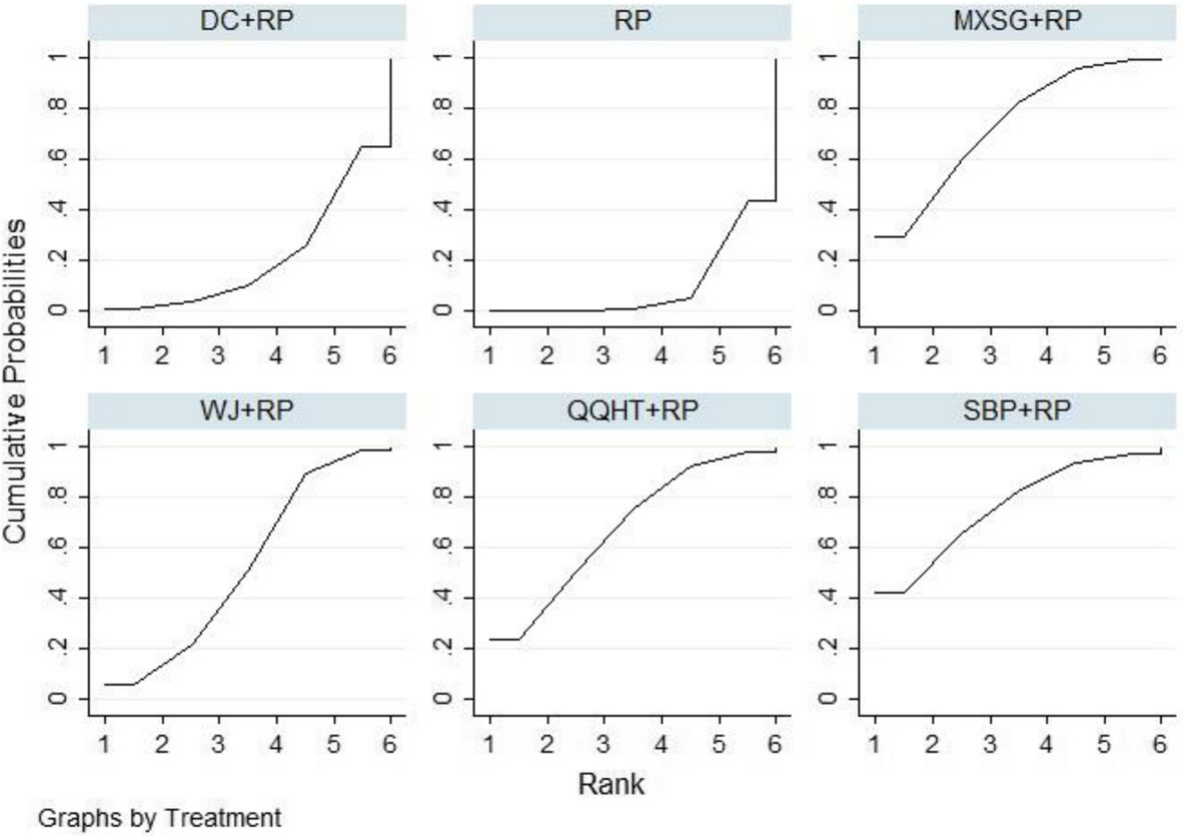
SUCRA for FEV1

Eighteen studies involving 1432 participants evaluated the FEV1 of lung function among the five Chinese herb formulas. The pair-wise meta-analysis showed that only two formulas demonstrated superior effects, compared to the use of routine pharmacotherapy alone (WJ: MD 0.25, 95%CI (0.19, 0.30); QQHT: MD 0.34, 95%CI (0.10, 0.58)) (Table 2). Network plots of the eligible comparisons for FEV1 are shown in Fig. 3. The results of the network meta-analysis revealed that SBP and MXSG were ranked the best in terms of FEV1 (Table 3). However, the results should be interpreted with caution because the significant differences among these formulas were not detected. The SUCRA is presented in Fig. 4.

**Table 2:** Pair-wised random-effects meta-analyses

Intervention	FEV1			PaO2			PaCO2			Effective rate		
	No. of studies	I/C	MD,95% CI	NO. of studies	I/C	MD,95% CI	NO. of studies	I/C	MD,95% CI	NO. of studies	I/C	OR,95% CI
WJ + RP vs RP	6	212/202	0.25[0.19,0.30]	8	270/260	9.90[5.02,14.78]	8	270/260	−5.40[− 8.33,-2.46]	10	344/322	3.60[2.32,5.59]
SBP + RP vs RP	2	79/80	0.40[−0.63,1.43]	5	154/154	4.24[1.10,7.38]	5	154/154	−5.33[−9.68,-0.99]	11	426/423	3.56[2.02,6.29]
YBBX + RP vs RP	0	NA	NA	0	NA	NA	0	NA	NA	2	88/88	2.26[0.85,5.99]
DC + RP vs RP	4	136/130	0.05[− 0.04,0.15]	1	42/40	5.20[−0.15.10.55]	1	42/40	−7.40[−12.17,-2.63]	6	211/205	3.63[1.91,6.89]
QQHT + RP vs RP	3	166/166	0.34[0.10,0.58]	3	92/92	11.74[4.21,19.27]	3	92/92	−7.65[−9.34,-5.95]	7	222/222	4.29[2.18,8.46]
MXSG + RP vs RP	3	131/129	0.38[−0.05,0.80]	3	130/130	6.66[−0.55,13.86]	2	80/80	−8.80[−10.21,-7.38]	13	683/669	2.77[1.86,4.11]

I/C: Sample size of intervention/control groups. *MD* Mean Difference, *CI* Confidence interval, *OR* Odds Ratio

**Table 3:**
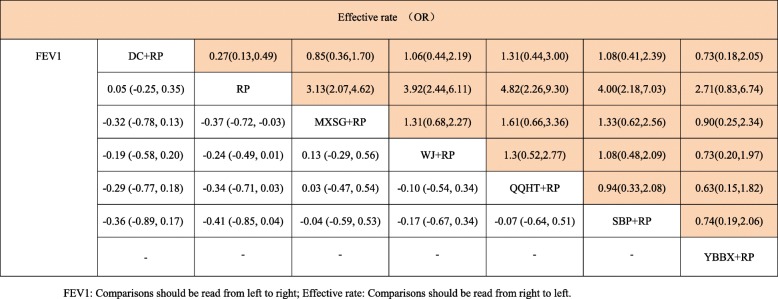
Network meta-analysis of FEV1 and Effective rate

#### PaO2 and PaCO2

**Fig. 5:**
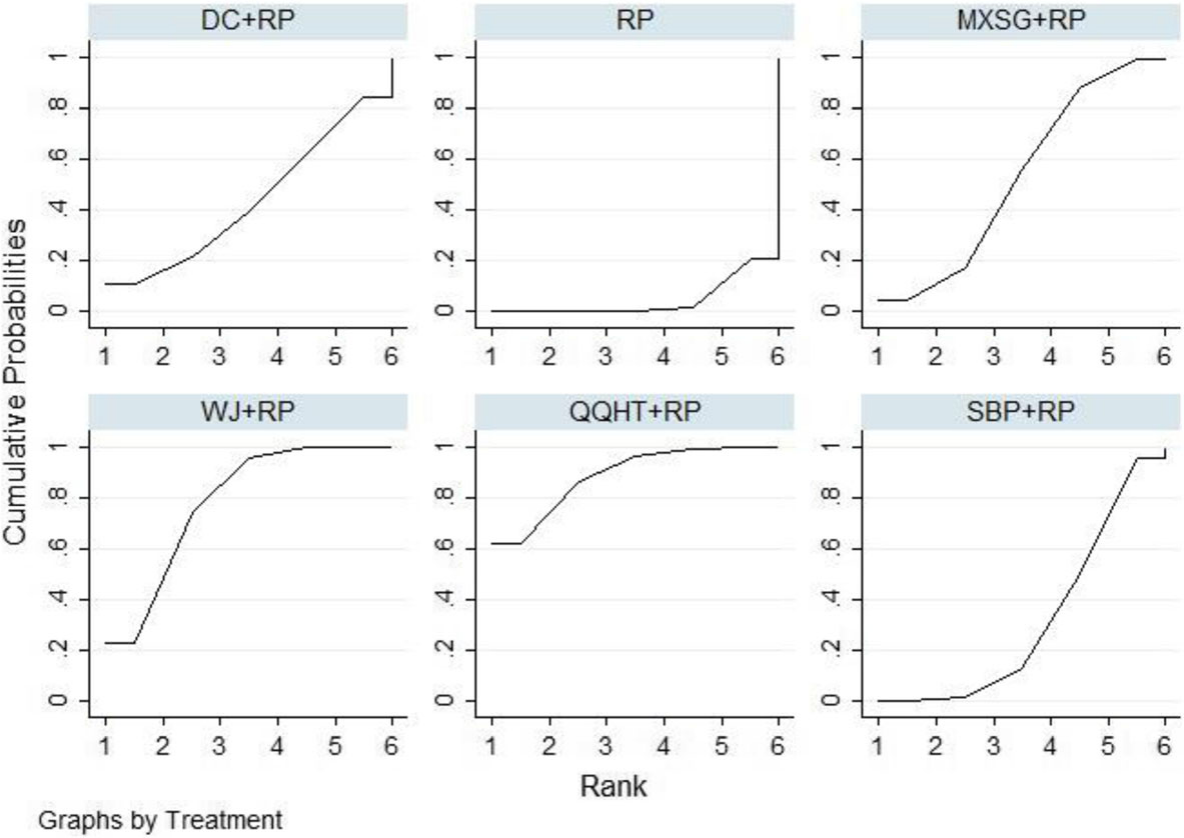
SUCRA for PaO2

**Fig. 6:**
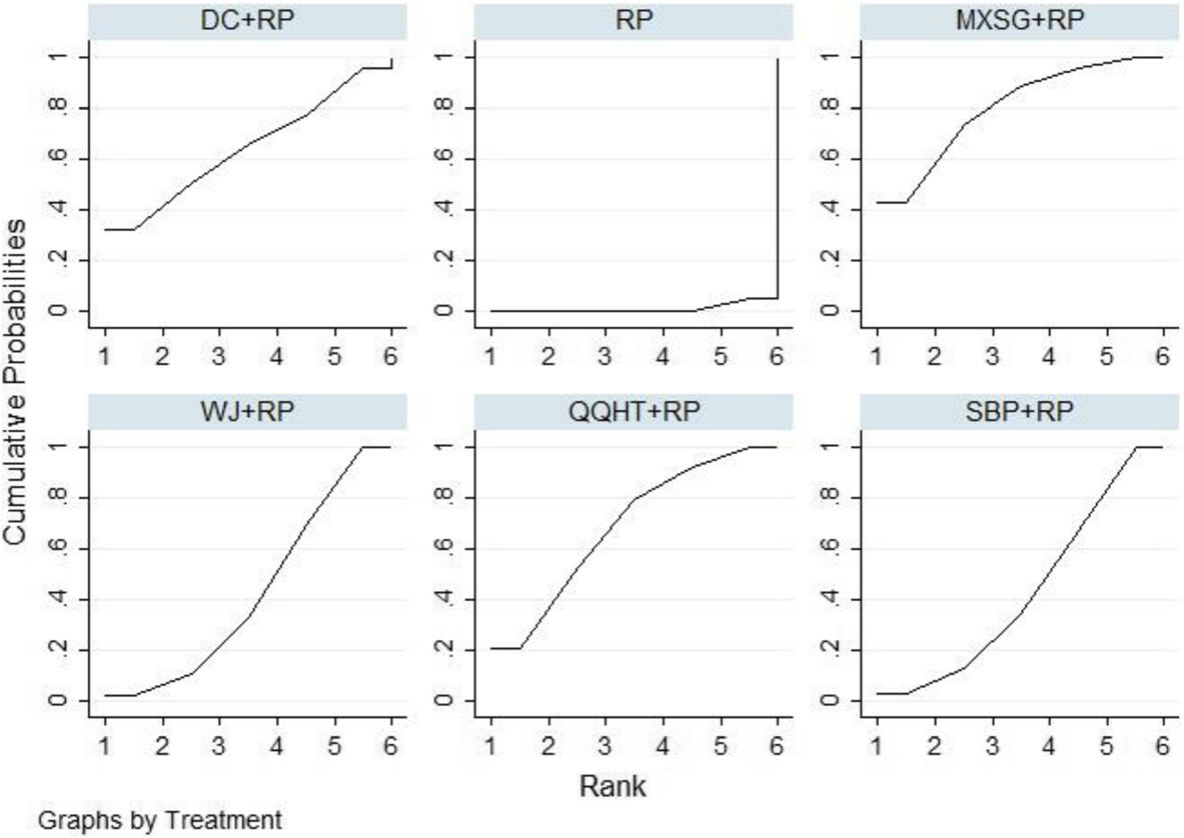
SUCRA for PaCO2

In terms of arterial blood gases, both PaO2 and PaCO2 were evaluated among the five Chinese herb formulas. The beneficial effects on both outcomes were observed in the SBP, WJ and QQHT formulas by the use of the pair-wise meta-analyses (Table 2). No significant changes were observed in PaO2 for MXSG and DC. The results of the NMA indicated that QQHT and WJ were more effective than SBP for improving PaO2. QQHT, WJ and MXSG may be optimal options for improving these two outcomes (Table 4, Figs. 5 and 6).

**Table 4:**
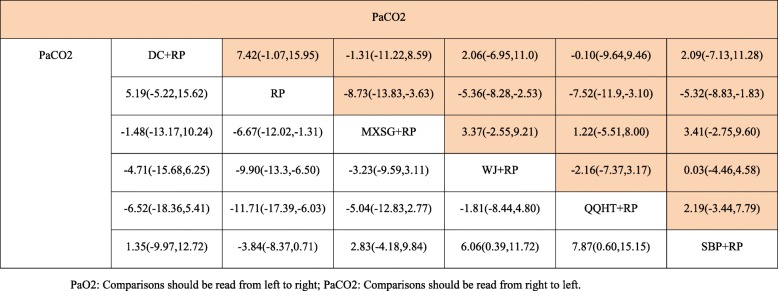
Network meta-analysis of PaO2 and PaCO2

#### Effective rate

**Fig. 7:**
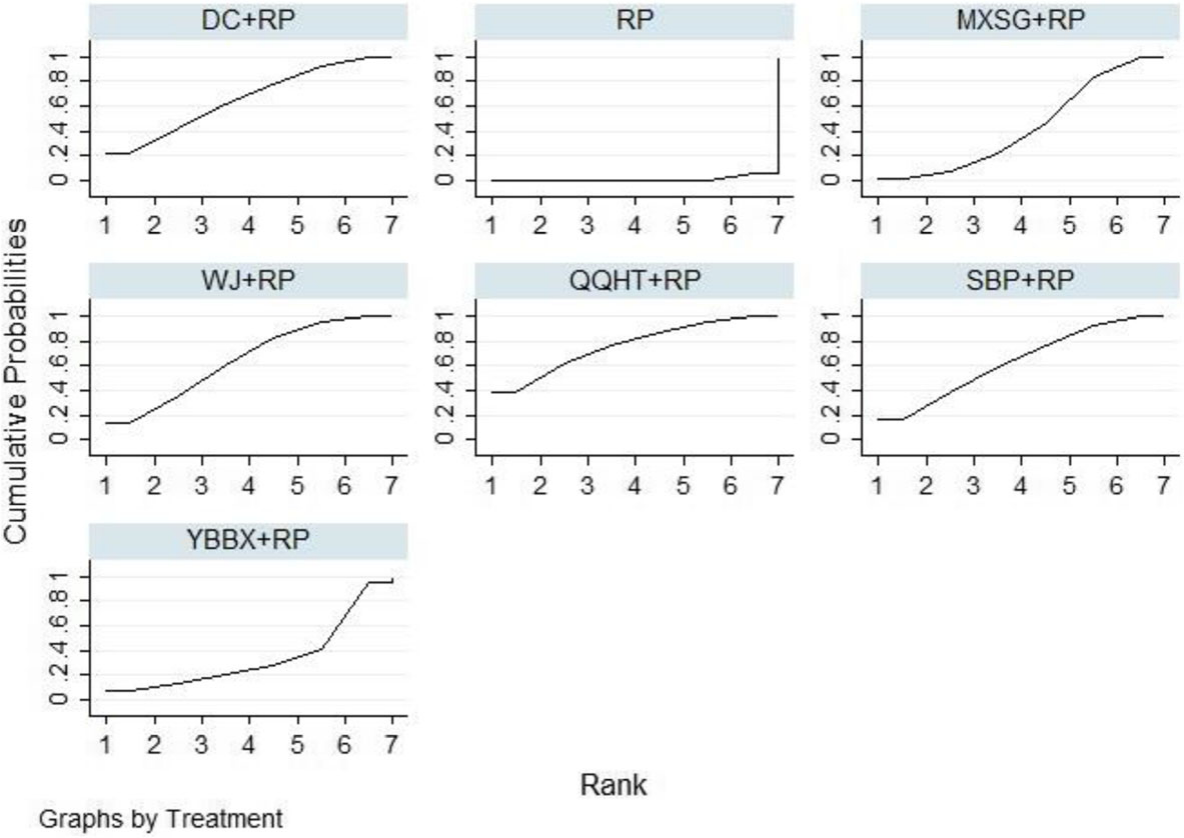
SUCRA for effective rate

A total of 47 studies were included in the comparisons of the effective rates among these six formulas. The effective rate was reported according to the Chinese medicine clinical research guidelines [19]. The criteria for the effective rate were not able to be verified in five studies [38, 39, 44, 64, 68]. These data were excluded from the analysis. The treatment effects that were estimated with the pair-wise random-effect meta-analysis showed that five of the six treatments had add-on favourable effects, compared to routine pharmacotherapy (WJ: OR 3.60, 95%CI (2.32,5.59); MXSG: OR 2.77, 95%CI (1.86,4.11); QQHT: OR 4.29, 95%CI (2.18,8.46); DC: OR 3.63, 95%CI (1.91,6.89); SBP: OR 3.56, 95%CI (2.02,6.29) and YBBX: OR 2.26, 95%CI (0.85,5.99)) (Table 2). Network diagrams of the eligible comparisons are presented in Fig. 3. The results of the network meta-analysis showed significant differences in the five formulas, when compared to the use of pharmacotherapy alone (OR 3.13, 95%CrI (2.07,4.62) for MXSG; OR 3.92, 95%CrI (2.44,6.11) for WJ; OR 4.82, 95%CrI (2.26,9.30) for QQHT; OR 4.00, 95%CrI (2.18,7.03) for SBP and OR 3.70, 95%CrI (2.04,7.69) for DC) (Table 3). The assessment for the YBBX formula did not demonstrate a significant difference (OR 2.71, 95%CrI (0.83, 6.74)). The efficacy ranking revealed that QQHT was the best treatment for the effective rate (Fig. 7).

#### Safety

Twenty-three studies reported no adverse events. One trial that evaluated the WJ formula reported mild events between the groups: liver and kidney dysfunction (4 cases) and indigestion (6 cases) in the treatment group, and liver and kidney dysfunction (4 cases) and indigestion (4 cases) in the control group [26]. Mild digestive dysfunction was observed in two studies that assessed the efficacy of SBP [24, 70]. The causalities of these adverse events were not explored.

#### Sensitivity analysis and publication bias

A sensitivity analysis was performed by removing the trials that did not report appropriate methods for random sequence generations. Nineteen studies of the six Chinese herb formulas that evaluated the effective rate were included, and the results did not show major changes when comparing the results with the overall estimates (Additional file 5). QQHT and WJ may have better effects among these formulas, in terms of the effective rate. A further analysis for the other outcomes was not conducted, due to insufficient data. The publication bias was assessed in the studies that reported the effective rates. A visual inspection of the funnel plot showed that potential biases may have existed among these studies (Additional file 6).

## Discussion

This network meta-analysis was conducted to compare the effects of six Chinese herb formulas combined with pharmacotherapy for the treatment of AECOPD. The results suggested that SBP and MXSG, as well as QQHT, appeared to be more effective for lung function improvement. QQHT, WJ and MXSG exhibited more favourable effects in terms of arterial blood gases (PaO2 and PaCO2). The effective rate was the sole outcome in evaluating all six of the Chinese herb formulas. The results indicated that QQHT may be the most effective formula, and consistent results were demonstrated in the sensitivity analysis. In summary, QQHT and MXSG might be the most effective formulas for the management of AECOPD.

For lung function, the results of the current pair-wise meta-analysis showed that only WJ and QQHT were observed to have significant changes on FEV1. In addition, only MXSG demonstrated a significant improvement in FEV1 compared with pharmacotherapy in the network meta-analysis. The possible reasons for these contradictory results are the small sample sizes of the included studies and the high heterogeneity that was observed among these studies. The results with wide confidence intervals may have changed from beneficial to non-beneficial if more studies were included. In addition, the NMA results showed that there was no significant difference among all five of the formulas. In addition to the small sample sizes of the included studies, another possible explanation was the similarity of the herb ingredients of these five formulas, such as *Ku Xing Ren* (*Semen Armeniacae Amarum*), *Ban Xia* (*Pinelliae Rhizoma*) and *Huang Qin* (*Scutellariae Radix*). These formulas or the active compositions of the herb ingredients have also shown common effects on anti-inflammation, anti-oxidative stress and immune function enhancement [75–78]. However, the ranking probabilities of these formulas could be explored according to the SUCRA. SBP, MXSG and QQHT seemed to be more effective than the other two formulas.

### Strengths and limitations

This study is the first comprehensive, systematic review to compare Chinese herb formulas for the treatment of AECOPD. The ranking of the commonly used formulas might be beneficial for clinical practice. Additionally, our study had some limitations. First, there were no head-to-head comparisons performed among these formulas. Second, some of the formulas were estimated by using small sample sizes and wide credible intervals, conclusions based on these studies are more likely to be changed by further research. Third, the formula ingredients of some studies varied substantially comparing with the fixed classical formulas, which may be difficult to replicate the results for other researchers. In addition, little information was reported for the use of routine pharmacotherapy among the included studies. In fact, the treatments also varied among the studies, in terms of antibiotic types, bronchodilators, doses and administration frequencies of these drugs. Several previous systematic reviews have compared the effectiveness of different treatment scenarios and have indicated different therapeutic advantages [79–81]. These differences might have introduced heterogeneity into our network meta-analysis. Therefore, the results should be interpreted with caution. Fourth, FEV1 as the primary outcome was only reported in eighteen studies and was evaluated in the five formulas. The results were still unreliable due to the small sample size. When considering that this outcome could provide objective measurements on the severity of the condition, future studies are suggested to include this outcome in order to reflect the clinically important outcomes. At last, methodology limitations existed in the included studies, such as inappropriate sequence generations, a lack of allocation concealment and a lack of blinding. Although we conducted a sensitivity analysis that focused on studies with low risks of bias on randomization, in order to explore the robustness of the results, the potential biases may affect the real therapeutic effects of these formulas due to the lack of blinding.

### Implications for research

Inadequate reporting in the current Chinese herb formula studies has affected the judgements about the efficacy and safety of Chinese Medicine (CM). For Chinese medicinal herbs, some may be referring to the multiple species [82, 83]. The absence of the herbal scientific names will introduce the difficulties to researchers in determining which kinds of species were observed. Moreover, this incompleteness may impede dissemination of information from clinical trials of CHM formulas and discourage the clinical practice [84]. Future studies of Chinese herb formulas should comply with the recommendations of the CONSORT Extension for Chinese herbal medicine formulas [84, 85]. Efforts on the standardized reporting of clinical trials will promote data quality, research transparency, repeatability, generalization and readiness for synthesis, and these efforts may also help the authors in minimizing biases in research design from the start of a study. Moreover, in terms of comparing the safety of a Chinese herb formula, observational studies are also encouraged, especially real-world studies that use electrical medical records with long-term durations of follow-up.

In addition to the heterogeneity of the Chinese herb formulas, another major challenge in evaluating the clinical effects is the variety of the uses of pharmacotherapy in different clinical and research settings. Hence, future treatment scenarios should strictly follow the clinical guidelines, which will be beneficial in identifying the optimal treatments for guiding clinical practice.

Further research should be designed with the use of rigorous methodology, and the key outcome measures need to be reported, especially for FEV1, lengths of hospital stay and mortality. The effective rate, as a composite outcome, is defined as the improvement of clinical symptoms, including cough, phlegm and dyspnoea. However, inconsistent results are always produced, due to a lack of objective criteria; thus, clinical treatment effectiveness should be evaluated with the use of better validated outcome measures.

## Conclusions

This comprehensive network meta-analysis showed that QQHT and MXSG might be the optimal Chinese herb formulas to combine with pharmacotherapy for the treatment of acute exacerbations of COPD. These six Chinese herb formulas seem to be similarly effective and significantly more effective than the use of pharmacotherapy alone in improvements of arterial blood gases and effective rates. However, the robustness of our findings may be affected due to the methodological limitations of the included studies. Further rigorous studies that include direct comparisons for these Chinese herb formulas, such as comparative effectiveness research, are encouraged to provide the most promising evidence for patients with AECOPD.

## Additional files

Additional file 1: The PRISMA-NMA checklist. (DOCX 29 kb)

Additional file 2: Search terms. (DOCX 18 kb)

Additional file 3: Summary of Chinese herb formulas. (DOCX 22 kb)

Additional file 4: Herb details in included studies. (DOCX 26 kb)

Additional file 5: Sensitivity analysis for effective rate. (PDF 1212 kb)

Additional file 6: Funnel plot of effective rate. (PDF 15 kb)

## Data Availability

All data used in this review are included in this published article.

## Abbreviations

AECOPD: Acute exacerbation of chronic obstructive pulmonary disease
AMED: Allied and Complementary Medicine Database
CBM: Chinese Biomedical Database
CENTRAL: Cochrane Central Register of Controlled Trials
CNKI: Chinese National Knowledge Infrastructure
COPD: Chronic obstructive pulmonary disease
CQVIP: Chongqing VIP Database
DC: Dingchuan decoction
MXSG: Maxingshigan decoction
OR: Odds ratio
PRISMA: Preferred Reporting Items for Systematic Reviews and Meta-Analysis
QQHT: Qingqihuatan decoction
SBP: Sangbaipi decoction
SUCRA: Surface under the cumulative ranking curve
WJ: Weijing decoction
YBBX: Yuebijiabanxia decoction

## References

[1] https://www.who.int/respiratory/copd/burden/en/. Accessed 7 Jan 2019.

[2] Soler-Cataluna JJ, Martinez-Garcia MA, Roman Sanchez P, Salcedo E, Navarro M, Ochando R (2005). Severe acute exacerbations and mortality in patients with chronic obstructive pulmonary disease. Thorax..

[3] Suissa S, Dell'Aniello S, Ernst P (2012). Long-term natural history of chronic obstructive pulmonary disease: severe exacerbations and mortality. Thorax..

[4] Ko FW, Chan KP, Hui DS, Goddard JR, Shaw JG, Reid DW (2016). Acute exacerbation of COPD. Respirology..

[5] Chinese Academy of Chinese Medical Sciences (2011). Evidence-Based Guidelines of Clinical Practice in Chinese Medicine.

[6] China Association of Chinese Medicine (2008). Guideline for diagnosis and treatment of common internal disease in Chinese medicine: Symptoms in Chinese medicine.

[7] China Association of Chinese Medicine (2018). Guideline for diagnosis and treatment of common internal disease in Chinese medicine: Diseases of modern medicine.

[8] Liu S, Shergis J, Chen X, Yu X, Guo X, Zhang AL (2014). Chinese herbal medicine (weijing decoction) combined with pharmacotherapy for the treatment of acute exacerbations of chronic obstructive pulmonary disease. Evid Based Complement Alternat Med.

[9] Yang X, Yang Z, Huang F, Zhang Y, Jiang J (2016). Therapeutic effect and symptom changes of phlegm-clearing decoction combined with western medicine for COPD with acute exacerbation: a systematic review and meta-analysis. Chin Archives of Tradit Chin Med.

[10] Jiang L, Gao M, Qu F, Li HL, Yu LB, Rao Y (2015). Pharmacokinetics of maxing Shigan decoction in normal rats and RSV pneumonia model rats by HPLC-MS/MS. Zhongguo Zhong Yao Za Zhi.

[11] Li L, Yu CH, Ying HZ, Yu JM (2013). Antiviral effects of modified dingchuan decoction against respiratory syncytial virus infection in vitro and in an immunosuppressive mouse model. J Ethnopharmacol.

[12] Li L, Bao H, Wu J, Duan X, Liu B, Sun J (2012). Baicalin is anti-inflammatory in cigarette smoke-induced inflammatory models in vivo and in vitro: a possible role for HDAC2 activity. Int Immunopharmacol.

[13] Cipriani A, Higgins JP, Geddes JR, Salanti G (2013). Conceptual and technical challenges in network meta-analysis. Ann Intern Med.

[14] Jansen JP, Crawford B, Bergman G, Stam W (2008). Bayesian meta-analysis of multiple treatment comparisons: an introduction to mixed treatment comparisons. Value Health.

[15] Salanti G, Ades AE, Ioannidis JP (2011). Graphical methods and numerical summaries for presenting results from multiple-treatment meta-analysis: an overview and tutorial. J Clin Epidemiol.

[16] Liu S, Chen J, He Y, Wu L, Lai J, Zuo J (2017). Comparative effectiveness of six Chinese herb formulas for acute exacerbation of chronic obstructive pulmonary disease: protocol for systematic review and network meta-analysis. BMJ Open.

[17] Hutton B, Salanti G, Caldwell DM, Chaimani A, Schmid CH, Cameron C (2015). The PRISMA extension statement for reporting of systematic reviews incorporating network meta-analyses of health care interventions: checklist and explanations. Ann Intern Med.

[18] Global Strategy for the Diagnosis, Management and Prevention of COPD, Global Initiative for Chronic Obstructive Lung Disease (GOLD) 2016. https://goldcopd.org/global-strategy-diagnosis-management-prevention-copd-2016/. Accessed 7 Jan 2019.

[19] Zheng X (2002). Guidance for clinical research on new drugs of TCM.

[20] Zou H (2015). To observe the clinical effect of integrated traditional and western medicine on chronic obstructive pulmonary disease at acute exacerbation stage. Shanxi J Tradit Chin Med.

[21] Zhou Z, Wei Y (2016). Efficacy of Maxingshigan decoction in treatment of chronic obstructive pulmonary disease with acute exacerbation and effects on inflammatory factor. Eval Anal Drug Hosp China.

[22] Zhou Y (2015). The academic ideas and clinical experiences of Prof. He and the clinical research of He Jia Wei Dingchuan decoction in the treatment of AECOPD.

[23] Zhou K (2016). Effect of Maxingshigan decoction combined with western medicine for the treatment of chronic obstructive pulmonary disease. Health.

[24] Zheng X (2014). Clinical efficacy on Jiawei Sangbaipi decoction in treating AECOPD with phlegm heat and blood stasis type and its influence on HMGB1 and FIB.

[25] Zhao W (2007). Effect of SOD and LPO in the peripheral blood and clinical research on efficacy of acute exacerbations of chronic obstructive pulmonary disease (AECOPD) with the management of clearing away heat-evil and resolving phlegm.

[26] Zhang L (2011). Effect of modified Weijing decoction for the treatment of acute exacerbations of chronic obstructive pulmonary disease. J Shandong Univ TCM.

[27] Zhang J (2012). Effect of Qingqihuatan decoction combined with routine pharmacotherapy for 30 cases of acute exacerbations of chronic obstructive pulmonary disease. Tradit Chin Med Res.

[28] Zhang J (2011). Clinical research of Maxingshigan decoction for the treatment of chronic obstructive pulmonary disease.

[29] Zhang J (2006). The clinical effect research and influence and meanings to IL-8 of the Weijing decoction of AECOPD.

[30] Zhang C, Mao J, Yang Q, Chen Y (2012). Clinical effect of integrative medicine for the treatment of syndrome of phlegm-heat obstructing lung for acute exacerbation of chronic obstructive pulmonary disease. Medical J Chin Peoples Health.

[31] Zhang C, Li D, Chen Z (2016). Clinical observation of Yuebijiabanxia decoction for the acute exacerbations of chronic obstructive pulmonary disease. Guangxi J Tradit Chin Med.

[32] Ye L, Hong X (2011). Effective observation of treating acute exacerbation of chronic obstructive pulmonary disease with Sangbaipi decoction. J Practical Tradit Chin Intern Med..

[33] Yang H. Clinical effect of Maxingshigan decoction for the treatment of syndrome of phlegm-heat obstructing lung of acute exacerbation of chronic obstructive pulmonary disease. Psychol Doct. 2012;306.

[34] Xie W (2009). Effect of modified Maxingshigan decoction for the treatment of acute exacerbation of chronic obstructive pulmonary disease. Chin Med Mod Distance Educ China.

[35] Xie J, Zeng X, Lin G (2011). Combine traditional Chinese and western medicine treatment of acute exacerbation of chronic obstructive pulmonary disease clinical observation period. J Liaoning Univ Tradit Chin Med.

[36] Wang X (2015). Maxingshigan decoction for treating AECOPD in 39 cases and nursing measures. China Pharm.

[37] Wang P, Lin X (2012). Clinical effect of Yuebijiabanxia decoction for the treatment of syndrome of phlegm-heat obstructing lung of acute exacerbation of chronic obstructive pulmonary disease. J Front of Med.

[38] Wang C, Guo W, Zhang F, Liu Y (2015). Clinical observation of Maxingshigan decoction for the treatment of acute exacerbation of chronic obstructive pulmonary disease and influence on inflammatory markers. World Latest Med Inf.

[39] Wang B (2016). Observation of traditional Chinese and western medicine treatment of acute exacerbation of chronic obstructive pulmonary disease. World Latest Med Inf.

[40] Sun X, Xu G (2015). Clinical observation on modified Maxingshigan decoction on treating acute exacerbations of chronic obstructive pulmonary disease. World Chin Med.

[41] Sun J (2012). The effect of Dilong Dingchuan decoction combined with western medicine for 31 cases of acute exacerbations of chronic obstructive pulmonary disease. Shandong J Tradit Chin Med.

[42] Shi Y (2005). Clinical research of Weijingxuanbi decoction for the treatment of acute exacerbations of chronic obstructive pulmonary disease.

[43] Ma D, Cai W (2013). Sangbaipi decoction on acute exacerbation of chronic obstructive pulmonary disease (phlegm-heat obstructing lung) clinical efficacy and prognosis. J Zhejiang Univ Tradit Chin Med.

[44] Lv T (2014). Clinical observation of Qingqihuatan decoction combined with western medicine for the treatment of acute exacerbations of chronic obstructive pulmonary disease. J Emerg in Tradit Chin Med..

[45] Liu X (2011). The clinical observation of acute onset of chronic obstructive pulmonary disease by Dingchuantang add-subtract atomization therapy.

[46] Liu J, Jing X, Liu X (2006). Influence of modified Qianjin Weijing decoction on airway clearance in patients with acutely exacerbated chronic obstructive pulmonary disease. J Guangzhou University of Tradit Chin Med.

[47] Lin Y, Ke J (2014). Effect of integrative medicine for 102 case of acute exacerbations of chronic obstructive pulmonary disease. Fujian J Tradit Chin Med..

[48] Lin J (2011). Clinical observation of modified Dingchuan decoction for acute exacerbation of chronic obstructive pulmonary disease. J New Chin Med.

[49] Li Z, Liu X, Li L, Ye Y, Zheng X (2016). Clinical observation of Sangbaipi decoction for syndrome of phlegm-heat obstructing lung of acute exacerbation of chronic obstructive pulmonary disease. J New Chin Med..

[50] Li Y (2012). Clinical research of modified Sangbaipi decoction for syndrome of phlegm-heat obstructing lung of acute exacerbations of chronic obstructive pulmonary disease.

[51] Li Y, Huang Z (2013). Clinical observation of treatment of 30 cases for acute exacerbations of chronic obstructive pulmonary disease by clearing heat and resolving phlegm. Hunan J Tradit Chin Med.

[52] Li X (2014). Clinical effect of medium frequency iontophoresis with Sangbaipi decoction for syndrome of phlegm-heat obstructing lung of acute exacerbations of chronic obstructive pulmonary disease.

[53] Li S, He Q, Cai Y (2013). Study on mechanism of Qingqihuatan decoction in treatment of acute exacerbation of chronic obstructive pulmonary disease by inflammatory cytokines. Chinese J Inf Tradit Chin Med.

[54] Li L (2016). Clinical effect of Maxingshigan decoction combined with western medicine for the treatment of acute exacerbations of chronic obstructive pulmonary disease. Chin J Clin Ration Drug Use.

[55] Li H (2012). The clinical observation of Qingqihuatan decoction treating phlegm-heat obstructing lung type of AECOPD.

[56] Ju P. Clinical observation of modified Qingqihuatan decoction for the treatment of syndrome of phlegm-heat obstructing lung of acute exacerbations of chronic obstructive pulmonary disease. Asia-pacific Tradit Med. 2015;11:103–5.

[57] Jing X (2011). Clinical research of modified Maxingshigan decoction for the treatment of acute exacerbations of chronic obstructive pulmonary disease.

[58] Jing X, Wang D, Li H, Cui X, Gao J (2009). Effects of inflammatory response and oxidative stress for Qingrehuatan prescription on acute exacerbations of chronic obstructive pulmonary disease. Chinese J Clinicians.

[59] Jing X, Li X, Liu J, Cui X (2007). Effect of modified Qianjin Weijing decoction on nuclear factor-kB of chronic obstructive pulmonary disease in acute deteriorated stage. Chin J TCM WM Crit care.

[60] Jiang H (2015). Effect analysis on integrated traditional Chinese and western medicine in the treatment of chronic obstructive pulmonary disease. China Modern Med.

[61] Jia J, Zhao H (2016). Clinical observation of Qingqihuatan decoction for syndrome of phlegm-heat obstructing lung of acute exacerbations of chronic obstructive pulmonary disease. J Emerg in Tradit Chin Med.

[62] Huang X (2013). White mulberry soup combined western medicine therapy with thermal yu lung phlegm random parallel control study of chronic obstructive pulmonary disease. J Practical Tradit Chin Intern Med.

[63] Hua W (2017). Clinical observation of integrative medicine for the treatment of acute exacerbations of chronic obstructive pulmonary disease. J Pract Tradit Chin Med.

[64] Hu J, Shi S, Yang J, Liu G (2015). Effect on inflammatory response for TLRs of Maxingshigan decoction for chronic obstructive pulmonary disease. J Sichuan Tradit Chin Med..

[65] Guo Y (2010). Clinical research of Weijing decoction combined western medicine for the treatment of acute exacerbations of chronic obstructive pulmonary disease. Hebei J Tradit Chin Med.

[66] Guo F, Fu L, Zhao B, Jiao C, Fu Y, Shi Y (2012). Clinical observation of modified Sangbaipi decoction combined routine care for syndrome of phlegm-heat obstructing lung for acute exacerbations of chronic obstructive pulmonary disease. Clinical J TCM.

[67] Gao X, Shen Y, Gong X (2017). Clinical research of modified Dingchuan decoction for 40 cases of phlegm-heat obstructing lung type of chronic obstructive pulmonary disease. Asia Pac Tradit Med.

[68] Fan H (2003). Effect of modified Qingqihuatan decoction for 57 cases of acute exacerbations of chronic obstructive pulmonary disease. J Sichuan of Tradit Chin Med.

[69] Chen X, Wang R (2009). Clinical observation of treatment of 31 cases of severe chronic obstructive pulmonary disease by clearing away heat, resolving phlegm and activating blood circulation combined with western drugs. Jiangsu J Tradit Chin Med.

[70] Chen X (2016). Effect of Sangbaipi decoction combined moxifloxacin for 65 cases of phlegm-heat obstructing lung type chronic obstructive pulmonary disease. Fujian J Tradit Chin Med.

[71] Chen H, Qiu L. Clinical effect of Qianjin modified Weijing decoction for 60 cases of acute exacerbations of chronic obstructive pulmonary disease. J Front Med. 2012;122.

[72] Bi W, Xu J, Zhang H, Zhang P (2016). Effect of modified Dingchuan decoction combined triple atomization inhalation for acute exacerbations of chronic obstructive pulmonary disease. Mod J Integr Tradit Chin West Med.

[73] Jing X, Wang D, Li X, Liu J, Xiong Y (2006). Research of Qingfeihuatan quyu prescription on the treatment of chronic obstructive pulmonary disease in acute deteriorated stage. Chin J TCM WM Crit Care..

[74] Wang X (2010). The clinical observation on ingredients-adding Qingqihuatan liquid in treating acute exacerbation of chronic obstructive pulmonary disease with heat-phlegm obstructing lung and impact of airway mucus hypersecretion.

[75] Wang G, Mohammadtursun N, Lv Y, Zhang H, Sun J, Dong J (2018). Baicalin exerts anti-airway inflammation and anti-Remodelling effects in severe stage rat model of chronic obstructive pulmonary disease. Evid Based Complement Alternat Med.

[76] Liczbinski P, Bukowska B (2018). Molecular mechanism of amygdalin action in vitro: review of the latest research. Immunopharmacol Immunotoxicol.

[77] Du W, Su J, Ye D, Wang Y, Huang Q, Gong X (2016). Pinellia ternata attenuates mucus secretion and airway inflammation after inhaled corticosteroid withdrawal in COPD rats. Am J Chin Med.

[78] Lee MY, Shin IS, Jeon WY, Lim HS, Kim JH, Ha H (2013). Pinellia ternata Breitenbach attenuates ovalbumin-induced allergic airway inflammation and mucus secretion in a murine model of asthma. Immunopharmacol Immunotoxicol.

[79] Mills EJ, Druyts E, Ghement I, Puhan MA (2011). Pharmacotherapies for chronic obstructive pulmonary disease: a multiple treatment comparison meta-analysis. Clin Epidemiol.

[80] Cope S, Donohue JF, Jansen JP, Kraemer M, Capkun-Niggli G, Baldwin M (2013). Comparative efficacy of long-acting bronchodilators for COPD: a network meta-analysis. Respir Res.

[81] Tricco AC, Strifler L, Veroniki AA, Yazdi F, Khan PA, Scott A (2015). Comparative safety and effectiveness of long-acting inhaled agents for treating chronic obstructive pulmonary disease: a systematic review and network meta-analysis. BMJ Open.

[82] http://www.theplantlist.org/. Accessed 5 Mar 2019.

[83] Chinese Pharmacopoeia Commission (2015). Pharmacopoeia of the People’s Republic of China.

[84] Cheng Chung-wah, Wu Tai-xiang, Shang Hong-cai, Li You-ping, Altman Douglas G., Moher David, Bian Zhao-xiang (2017). CONSORT Extension for Chinese Herbal Medicine Formulas 2017: Recommendations, Explanation, and Elaboration. Annals of Internal Medicine.

[85] Ioannidis JP, Evans SJ, Gotzsche PC, O'Neill RT, Altman DG, Schulz K (2004). Better reporting of harms in randomized trials: an extension of the CONSORT statement. Ann Intern Med.

