# Supplementary material for: Comparative effectiveness of six Chinese herb formulas for acute exacerbation of chronic obstructive pulmonary disease: a systematic review and network meta-analysis
Source: BMC Complement Altern Med. 2019 Aug 22;19:226. doi: 10.1186/s12906-019-2633-2 (PMC6704718; doi:10.1186/s12906-019-2633-2)
Supplement: Supplementary file 2 — Search terms. (DOCX 18 kb) [file 12906_2019_2633_MOESM2_ESM.docx]

| Search block | Search terms |
| --- | --- |
| Participants | Pulmonary Disease, Chronic Obstructive OR Bronchitis, Chronic OR Pulmonary Emphysema OR Emphysema OR COPD OR Chronic Obstructive Pulmonary OR COAD OR Chronic Obstructive Airway OR Chronic Obstructive Lung OR Chronic obstructive bronchopulmonary OR Chronic obstructive respiratory OR Chronic Airflow Obstruction OR Chronic Airflow Obstructive OR Chronic bronchitis OR Pulmonary emphysema OR Lung emphysema OR Chronic Airflow limitation. |
| Intervention | wejing decoction OR wejing tang OR sangbaipi decoction OR sangbaipi tang OR maxingshigan decoction OR maxingshigan tang OR yuebijiabanxia decoction OR yuebijiabanxia tang OR dingchuan decoction OR dingchuan tang OR qingqihuatan decoction OR qingqihuatan tang OR qingqihuatan pill |
| Study design | Randomized controlled trial OR controlled clinical trial OR randomized OR placebo OR drug therapy OR randomly OR trial OR groups |
