# Supplementary material for: Comparative effectiveness of six Chinese herb formulas for acute exacerbation of chronic obstructive pulmonary disease: a systematic review and network meta-analysis
Source: BMC Complement Altern Med. 2019 Aug 22;19:226. doi: 10.1186/s12906-019-2633-2 (PMC6704718; doi:10.1186/s12906-019-2633-2)
Supplement: Supplementary file 3 — Summary of Chinese herb formulas. (DOCX 22 kb) [file 12906_2019_2633_MOESM3_ESM.docx]

| Formula name | Herb ingredients  (Pin Yin name)^a^ | Scientific name^b^ |
| --- | --- | --- |
| Weijing decoction (WJ) | *Wei Jing* | *Phragmites communis Trin.* |
|  | *Yi Yi Ren* | *Coix lacryma-jobi L. var. mayuen (Roman.) Stapf* |
|  | *Tao Ren* | *Prunus persica (L.) Batsch.; Prunus davidiana (Carr.) Franch.* |
|  | *Dong Gua Ren* | *Benincasa hispida (Thunb.) Cogn.* |
| Maxingshigan decoction (MXSG) | *Ma Huang* | *Ephedra sinica Stapf.; Ephedra equisetina Bge.; Ephedra intermedia Schrenk et C.A. Mey.* |
|  | *Ku Xing Ren* | *Prunus armeniaca L. var. ansu Maxim.; Prunus sibirica L.; Prunus mandshurica (Maxim.) Koehne.; Prunus armeniaca L.* |
|  | *Shi Gao* | *Hydrated calcium sulfate* |
|  | *Gan Cao* | *Glycyrrhiza uralensis Fisch.; Glycyrrhiza inflata Bat.; Glycyrrhiza glabra L.* |
| Yuebijiabanxia decoction (YBBX) | *Ma Huang* | *Ephedra sinica Stapf.; Ephedra equisetina Bge.; Ephedra intermedia Schrenk et C.A. Mey.* |
|  | *Shi Gao* | *Hydrated calcium sulfate* |
|  | *Sheng Jiang* | *Zingiber officinale Rosc.* |
|  | *Da Zao* | *Ziziphus jujuba Mill.* |
|  | *Gan Cao* | *Glycyrrhiza uralensis Fisch.; Glycyrrhiza inflata Bat.; Glycyrrhiza glabra L.* |
|  | *Ban Xia* | *Pinellia ternata (Thunb.) Breit.* |
| Qingqihuatan decoction (QQHT) | *Chen Pi* | *Citrus reticulata Blanco* |
|  | *Ku Xing Ren* | *Prunus armeniaca L. var. ansu Maxim.; Prunus sibirica L.; Prunus mandshurica (Maxim.) Koehne.; Prunus armeniaca L.* |
|  | *Zhi Shi* | *Citrus aurantium L.; Citrus sinensis Osbeck* |
|  | *Huang Qin* | *Scutellaria baicalensis Georgi* |
|  | *Gua Lou Ren* | *Trichosanthes kirilowii Maxim.; Trichosanthes rosthronii Harms* |
|  | *Fu Ling* | *Poria cocos (Schw.) Wolf* |
|  | *Dan Nan Xing* | *Arisaema erubescens (Wall.) Schott.; Arisaema heterophyllum Bl.; Arisaema amurense Maxim.* |
|  | *Ban Xia* | *Pinellia ternata (Thunb.) Breit.* |
| Dingchuan decoction (DC) | *Bai Guo* | *Ginkgo biloba L.* |
|  | *Ma Huang* | *Ephedra sinica Stapf.; Ephedra equisetina Bge.; Ephedra intermedia Schrenk et C.A. Mey.* |
|  | *Zi Su Zi* | *Perilla frutescens (L.) Britt.* |
|  | *Gan Cao* | *Glycyrrhiza uralensis Fisch.; Glycyrrhiza inflata Bat.; Glycyrrhiza glabra L.* |
|  | *Kuan Dong Hua* | *Tussilago farfara L.* |
|  | *Ku Xing Ren* | *Prunus armeniaca L. var. ansu Maxim.; Prunus sibirica L.; Prunus mandshurica (Maxim.) Koehne.; Prunus armeniaca L.* |
|  | *Sang Bai Pi* | *Morus alba L.* |
|  | *Huang Qin* | *Scutellaria baicalensis Georgi* |
|  | *Fa Ban Xia* | *Pinellia ternata (Thunb.) Breit.* |
| Sangbaipi decoction (SBP) | *Sang Bai Pi* | *Morus alba L.* |
|  | *Ban Xia* | *Pinellia ternata (Thunb.) Breit.* |
|  | *Zi Su Zi* | *Perilla frutescens (L.) Britt.* |
|  | *Ku Xing Ren* | *Prunus armeniaca L. var. ansu Maxim.; Prunus sibirica L.; Prunus mandshurica (Maxim.) Koehne.; Prunus armeniaca L* |
|  | *Bei Mu* | *Fritillaria thunbergii Miq.* |
|  | *Zhi Zi* | *Gardenia jasminoides Ellis* |
|  | *Huang Qin* | *Scutellaria baicalensis Georgi* |
|  | *Huang Lian* | *Coptis chinensis Franch.; Coptis deltoidea C. Y. Cheng et Hsiao.; Coptis teeta Wall.* |

Note: Some herbs such as *Ephedra sinica* can be toxic and some have been classified as endangered by the IUCN (e.g. *Ginkgo biloba*). The applications of herbs are advised to comply with relevant regulations.

^a^Herb ingredients based on Great Compendium of Chinese Medical Formulae (Zhong Yi Fang Ji Da Ci Dian).

^b^Scientific names based on Pharmacopoeia of the People’s Republic of China 2015. Some herbs may refer to more than one species.
