# Supplementary material for: Comparative effectiveness of six Chinese herb formulas for acute exacerbation of chronic obstructive pulmonary disease: a systematic review and network meta-analysis
Source: BMC Complement Altern Med. 2019 Aug 22;19:226. doi: 10.1186/s12906-019-2633-2 (PMC6704718; doi:10.1186/s12906-019-2633-2)
Supplement: Supplementary file 4 — Herb details in included studies. (DOCX 26 kb) [file 12906_2019_2633_MOESM4_ESM.docx]

| Study ID | Formula | Herb ingredients and dosage (g) |
| --- | --- | --- |
| Zou HD 2015 | MXSG | *Ma Huang 5g, Chan Tui 5g, Yu Xing Cao 15g, Shi Gao 15g, Yi Yi Ren 15g, Chen Pi 15g, Ku Xing Ren 10g, Ren Shen 10g, Long Li Ye 10g, Gan Cao 10g, Ban Xia 10g, Bei Mu 10g, Dong Gua Ren 10g, Zi Su Zi 10g, Di Long 10g, Tao Ren 10g* |
| Zhou ZJ 2016 | MXSG | *Jie Geng 15g, Ku Xing Ren 15g, Ma Huang 15g, Gan Cao 10g, Da Huang 50g, Shi Gao 30g* |
| Zhou YH 2015 | DC | *Bai Bu, Ma Huang, Sang Bai Pi, Ku Xing Ren, Zi Wan, Bai Qian, Ban Xia, Ju Hong, Zi Su Zi, Huang Qin, Bei Mu, Kuan Dong Hua, Di Long (*dosage not specified*)* |
| Zhou KL 2016 | MXSG | *Ma Huang 7g, Shi Gao 15g, Ku Xing Ren 10g, Gan Cao 7g, Sang Bai Pi 12g, Ren Shen Ye 8g, Chen Pi 12g, Yu Xing Cao 12g, Chan Yi 6g, Ban Xia 8g* |
| Zheng XM 2014 | SBP | *Sang Bai Pi 15g, Huang Lian 3g, Huang Qin 6g, Gua Lou 10g, Bei Mu 9g, Zhi zi 6g, Ku Xing Ren 9g, Zi Su Zi 10g, Ban Xia 10g, Da Huang 3g, Dan Shen 15g, Yi Mu Cao 12g, Tao Ren 10g, Hou Po 9g* |
| Zhao WH 2007 | WJ | *Wei Jing 30g, Tao Ren 10g, Dong Gua Ren 30g, Yi Yi Ren 30g, Yu Xing Cao 30g, Ku Xing Ren 10g, Bei Mu15g, Hai Ge Qiao 30g, Zi Wan 10g, Kuan Dong Hua 10g, Gan Cao 6g* |
| Zhang LS 2011 | WJ | *Wei Jing 30g, Dong Gua Ren 20g, Yi Yi Ren 30g, Tao Ren30g, Huang Qin 30g, Gua Lou 15g, Bei Mu 15g, Jie Geng 12g* |
| Zhang JH 2012 | QQHT | *Chen Pi 6g, Ku Xing Ren 6g, Zhi Shi 6g, Huang Qin 6g, Gua Lou Ren 6g, Fu Ling 6g, Dan Nan Xing 9g, Ban Xia 9g* |
| Zhang JH 2006 | WJ | *Wei Jing 60g, Tao Ren 9g, Dong Gua Ren 24g, Yi Yi Ren 30g, Ku Xing Ren 10g, Bei Mu 15g, Hai Ge Qiao 30g, Yu Xing Cao 20g* |
| Zhang J 2011 | MXSG | *Shi Gao 30g, Ma Huang 9g, Ku Xing Ren 10g, Gan Cao 15g, Bei Mu 15g, Jie Geng 15g, Chen Pi 15g, Gua Lou 15g, Ting Li Zi 15g, Mai Dong 15g* |
| Zhang CM 2012 | SBP | *Sang Bai Pi 10g, Huang Qin 10g, Zhi zi 10g, Ku Xing Ren 10g, Ban Xia 10g, Bei Mu 10g, Zi Su Zi 10g, Jie Geng 10g, Gan Cao 6g* |
| Zhang CL 2016 | YBBX | *Ma Huang 9g, Shi Gao 21g, Ban Xia 9g, Gan Cao 6g, Sheng Jiang 6g, Da Zao 6g* |
| Ye L 2011 | SBP | *Sang Bai Pi 15g, Bei Mu 12g, Ku Xing Ren 6g, Huang Lian 3g, Zi Su Zi 10g, Ban Xia 10g, Huang Qin 9g, Zhi zi 9g* |
| Yang HW 2012 | MXSG | *Ma Huang, Ku Xing Ren, Shi Gao, Lian Qiao, Huang Qin, Sang Bai Pi, Di Long, Yu Xing Cao, Gan Cao (*dosage not specified*)* |
| Xie WH 2009 | MXSG | *Ma Huang 9g, Ku Xing Ren 10g, Shi Gao 20g, Zi Su Zi 10g, Di Long 12g, Huang Qin 12g, Dan Nan Xing 6g, Lian Qiao 15g, Ban Xia 10g, Gan Cao 10g* |
| Xie JJ 2011 | YBBX | *Ma Huang 6g, Shi Gao 20g, Ban Xia 10g, Sang Bai Pi 20g, Zhi Mu 20g, Zi Wan 20g, Kuan Dong Hua 20g, Zi Su Zi 10g, Ku Xing Ren 10g, Bei Mu 10g, Gan Cao 10g, Huang Qin 15g, Chen Pi 15g* |
| Wang XP 2015 | MXSG | *Ma Huang, Ku Xing Ren, Shi Gao, Gan Cao (*dosage not specified*)* |
| Wang PC 2012 | YBBX | *Ma Huang 10g, Shi Gao 30g, Sheng Jiang 6g, Da Zao 12g, Gan Cao 6g, Ban Xia 15g, Dan Shen 20g, Di Long 10g* |
| Wang CH 2015 | MXSG | *Ma Huang 6g, Ku Xing Ren 15g, Gan Cao 6g, Shi Gao 15g, Bei Mu 9g，Jin Qiao Mai 20g，Pi Pa Ye 15g* |
| Wang BH 2016 | WJ | *Wei Jing 15g, Dong Gua Ren 15g, Bei Mu 15g, Dan Shen 15g, Shan zha 15g, Yi Yi Ren 25g, Gua Lou Pi 10g, Zi Wan 10g, Kuan Dong Hua 10g, Tao Ren 10g, Zi Su Zi 10g* |
| Sun XS 2015 | MXSG | *Ma Huang 9g, Shi Gao 20g, Ku Xing Ren 15g, Da Huang 12g, Yu Xing Cao 15g, Sang Bai Pi 12g, Gua Lou 12g, Tao Ren 12g, Dang Gui 12g, Ban Xia 9g, Zi Su Zi 12g, Gan Cao 9g* |
| Sun JF 2012 | DC | *Di Long 10g, Ma Huang 5g, Sang Bai Pi 10g, Huang Qin 10g, Bei Mu 10g, Ku Xing Ren 10g, Tao Ren 10g, Zi Wan 10g, Kuan Dong Hua 10g, Ban Xia 10g, Ting Li Zi 10g, Gua Lou Pi 15g, Wu Wei Zi 10g, Gan Cao 10g* |
| Shi YY 2005 | WJ | *Wei Jing 30g, She Gan 12g, Pi Pa Ye 15g, Tao Ren 12g, Yu Jin 15g, Dong Gua Ren 30g, Yi Yi Ren 30g, Ku Xing Ren 12g, Hua Shi 15g, Huang Qin 15g, Gua Lou Ke 15g, Qian Hu 15g, Ting Li Zi 15g* |
| Ma DN 2013 | SBP | *Sang Bai Pi 12g, Ku Xing Ren 10g, Ban Xia 10g, Zi Su Zi 10g, Bei Mu 12g, Huang Qin 12g, Huang Lian 3g, Zhi zi 6g* |
| Lv T 2014 | QQHT | *Chen Pi 6g, Ku Xing Ren 6g, Zhi Shi 6g, Huang Qin 6g, Gua Lou Ren 6g, Fu Ling 6g, Dan Nan Xing 9g, Ban Xia 9g* |
| Liu X 2011 | DC | *Ma Huang 5g, Bai Guo 10g, Sang Bai Pi 15g, Huang Qin 15g, Ku Xing Ren 5g, Zi Su Zi 10g, Ban Xia 5g, Kuan Dong Hua 15g, Gan Cao 5g* |
| Liu JB 2006 | WJ | *Wei Jing, Yu Xing Cao, Dong Gua Ren , Yi Yi Ren , Tao Ren, Jie Geng, Gua Lou Ren, Bei Mu, Huang Qin* |
| Lin YZ 2014 | MXSG | *Ma Huang 6g, Ku Xing Ren 15g, Shi Gao 30g, Gan Cao 6g* |
| Lin J 2011 | DC | *Bai Guo 15g, Ma Huang 8g, Huang Qin 8g, Zi Su Zi 10g, Ku Xing Ren 10g, Sang Bai Pi 10g, Kuan Dong Hua 10g, Ban Xia 10g, Chen Pi 10g, Gan Cao 5g* |
| Li ZR 2016 | SBP | *Sang Bai Pi 15g, Bei Mu 15g, Ban Xia 10g, Zi Su Zi 10g, Huang Qin 10g, Ku Xing Ren 10g, Ma Huang 10g, Zhi Shi 10g, Gan Cao 6g* |
| Li YM 2012 | SBP | *Sang Bai Pi 10g, Huang Qin 10g, Zhi zi 10g, Da Huang 4g, Zi Su Zi 10g, Ku Xing Ren 10g, Bei Mu 10g, Ban Xia 10g, Gua Lou 20g, Fu Ling 10g* |
| Li Y 2013 | QQHT | *Dan Nan Xing 15g, Ban Xia 15g,Gua Lou Ren 10g, Huang Qin 10g, Chen Pi 10g, Ku Xing Ren 10g, Zhi Shi 10g, Fu Ling 10g* |
| Li XC 2014 | SBP | *Not specified* |
| Li SQ 2013 | QQHT | *Chen Pi 10g, Ku Xing Ren 10g, Zhi Shi 10g, Huang Qin 15g, Gua Lou Ren 15g, Fu Ling 20g, Dan Nan Xing 10g, Ban Xia 10g, Sheng Jiang 3片* |
| Li L 2016 | MXSG | *Shi Gao 30g, Ku Xing Ren 12g, Wei Jing 30g, Jie Geng 12g, Huang Qin 12g, Bei Mu 12g, Ma Huang 10g, Kuan Dong Hua 10g, Bai Bu 10g, Gan Cao 6g* |
| Li HM 2012 | QQHT | *Chen Pi 15g, Ku Xing Ren 15g, Zhi Shi 10g, Huang Qin 10g, Gua Lou 15g, Fu Ling 15g, Dan Nan Xing 10g, Ban Xia 10g, Jie Geng 20g, Zhi Mu 10g, Zhi zi 10g, Gan Cao 6g* |
| Ju P 2015 | QQHT | *Dan Nan Xing 10g, Huang Qin 12g, Gua Lou Ren 10g, Ban Xia 12g, Zhi Shi 10g, Chen Pi 12g, Fu Ling 15g, Ku Xing Ren 10g, Chi Shao 15g, Dan Shen 30g, Dang Shen 15g, Huang Qi 15g* |
| Jing X 2011 | MXSG | *Shi Gao 20g, Ma Huang 4g, Sang Bai Pi 10g, Yu Xing Cao 20g, Di Long 10g, Ban Xia lOg, Gua Lou 10g, Ku Xing Ren 10g, Pi Pa Ye 10g, Tao Ren 10g, Dang Gui 10g, Gan Cao 6g* |
| Jing XL 2009 | WJ | *Wei Jing 30g, Dong Gua Ren 20g, Yi Yi Ren 30g, Tao Ren 15g, Gua Lou 15g, Bei Mu 15g, Jie Geng 15g, Huang Qin 15g* |
| Jing XL 2007 | WJ | *Wei Jing 30g, Dong Gua Ren 20g, Yi Yi Ren 30g, Tao Ren 30g, Huang Qin 30g, Gua Lou 15g, Bei Mu 15g, Jie Geng 12g* |
| Jiang H 2015 | MXSG | *Ma Huang 6g, Ku Xing Ren 12g, Shi Gao 15g, Gan Cao 6g, Ren Shen Ye 9g, Long Li Ye 9g, Yu Xing Cao 15g, Chen Pi 12g, Ban Xia 9g, Chan Yi 5g* |
| Jia JY 2016 | QQHT | *Dan Nan Xing 9g, Gua Lou 20g, Huang Qin 10g, Ban Xia 9g, Ku Xing Ren 9g, Zhi Shi 10g, Chen Pi 9g, Fu Ling 15g, Sheng Jiang 6g* |
| Huang XB 2013 | SBP | *Zhi zi 9g, Ma Huang 10g, Gan Cao 10g, Ku Xing Ren 10g, Huang Lian 12g, Ban Xia 12g, Zi Su Zi 15g, Bei Mu 15g, Sang Bai Pi 15g, Huang Qin 15g, Shi Gao 30g* |
| Hua WS 2017 | MXSG | *Ma Huang 5g，Ku Xing Ren 10g，Shi Gao 20g，Huang Qin 12g，She Gan 12g，Jie Geng 12g，Zi Wan 12g，Kuan Dong Hua 12g，Hai Fu Shi 30g，Ting Li Zi 30g，Qian Hu 10g，Pi Pa Ye 20g* |
| Hu J 2015 | MXSG | *Not specified* |
| Guo YY 2010 | WJ | *Wei Jing 60g, Yi Yi Ren 30g, Dong Gua Ren 24g, Tao Ren 9g, Yu Xing Cao 30g* |
| Guo F 2012 | SBP | *Sang Bai Pi 15g, Huang Qin 10g, Huang Lian 6g, Zhi zi 12g, Sang Ye 15g, Ku Xing Ren 10g, Bei Mu 15g, Ban Xia 12g, Ting Li Zi 15g, Zi Su Zi 10g, Gua Lou 15g, Yu Xing Cao30g, Jie Geng 10g, Dan Shen 15g, Gan Cao 6g* |
| Gao X 2017 | DC | *Ma Huang 10g, Bai Guo 10g, Kuan Dong Hua 15g, Ban Xia 15g, Zi Su Zi 20g, Sang Bai Pi 10g, Huang Qin 20g, Ku Xing Ren 15g, Gan Cao 5g* |
| Fan HL 2003 | QQHT | *Dan Nan Xing 10g, Huang Qin 12g, Gua Lou 15g, Ban Xia 12g, Zhi Shi 10g, Chen Pi 12g, Fu Ling 15g, Yu Xing Cao 20g, Jin Yin Hua 20g ,Pu Gong Ying 20g, Chi Shao 15g, Dan Shen 30g, Huang Qi 20g* |
| Chen XM 2009 | WJ | *Wei Jing 30g, Tao Ren 15g, Yi Yi Ren 20g, Dong Gua Ren 20g* |
| Chen XP 2016 | SBP | *Sang Bai Pi 10g, Ban Xia 10g, Zi Su Zi 10g, Ku Xing Ren 10g, Bei Mu 12g, Huang Qin 12g, Huang Lian 3g, Zhi zi 5g, Di Long 10g, Gan Cao 5g* |
| Chen HY 2012 | WJ | *Wei Jing 30g, Tao Ren 15g, Dong Gua Ren 15g, Yi Yi Ren 15g* |
| Bi WZ 2016 | DC | *Huang Qi 30g, Sang Bai Pi 20g, Gan Cao 6g, Ma Huang 8g, Huang Qin 15g, Zi Wan 15g, Tai Zi Shen 15g, Ku Xing Ren 9g, Zhi Mu 15g, Zi Su Zi 9g, Bai Guo 15g, Kuan Dong Hua 15g* |
| Jing XL 2006 | WJ | *Wei Jing 30g, Dong Gua Ren 20g, Yi Yi Ren 30g, Tao Ren 30g, Huang Qin 30g, Gua Lou 15g, Bei Mu 15g, Jie Geng 12g* |
| Wang X 2010 | QQHT | *Huang Qin 15g, Zhi Shi 10g, Fu Ling 15g, Ban Xia 15g, Chen Pi 15g, Gua Lou 30g, Dan Nan Xing 15g, Ku Xing Ren 15g, Jie Geng 15g, Yu Xing Cao 30g, Pi Pa Ye 15g* |

WJ: Weijing decoction; SBP: Sangbaipi decoction; YBBX: Yuebijiabanxia decoction; DC: Dingchuan decoction; QQHT: Qingqihuatan decoction; MXSG: Maxingshigan decoction;
