# Supplementary figures and images for: Comparative effectiveness of six Chinese herb formulas for acute exacerbation of chronic obstructive pulmonary disease: a systematic review and network meta-analysis
Source: BMC Complement Altern Med. 2019 Aug 22;19:226. doi: 10.1186/s12906-019-2633-2 (PMC6704718; doi:10.1186/s12906-019-2633-2)

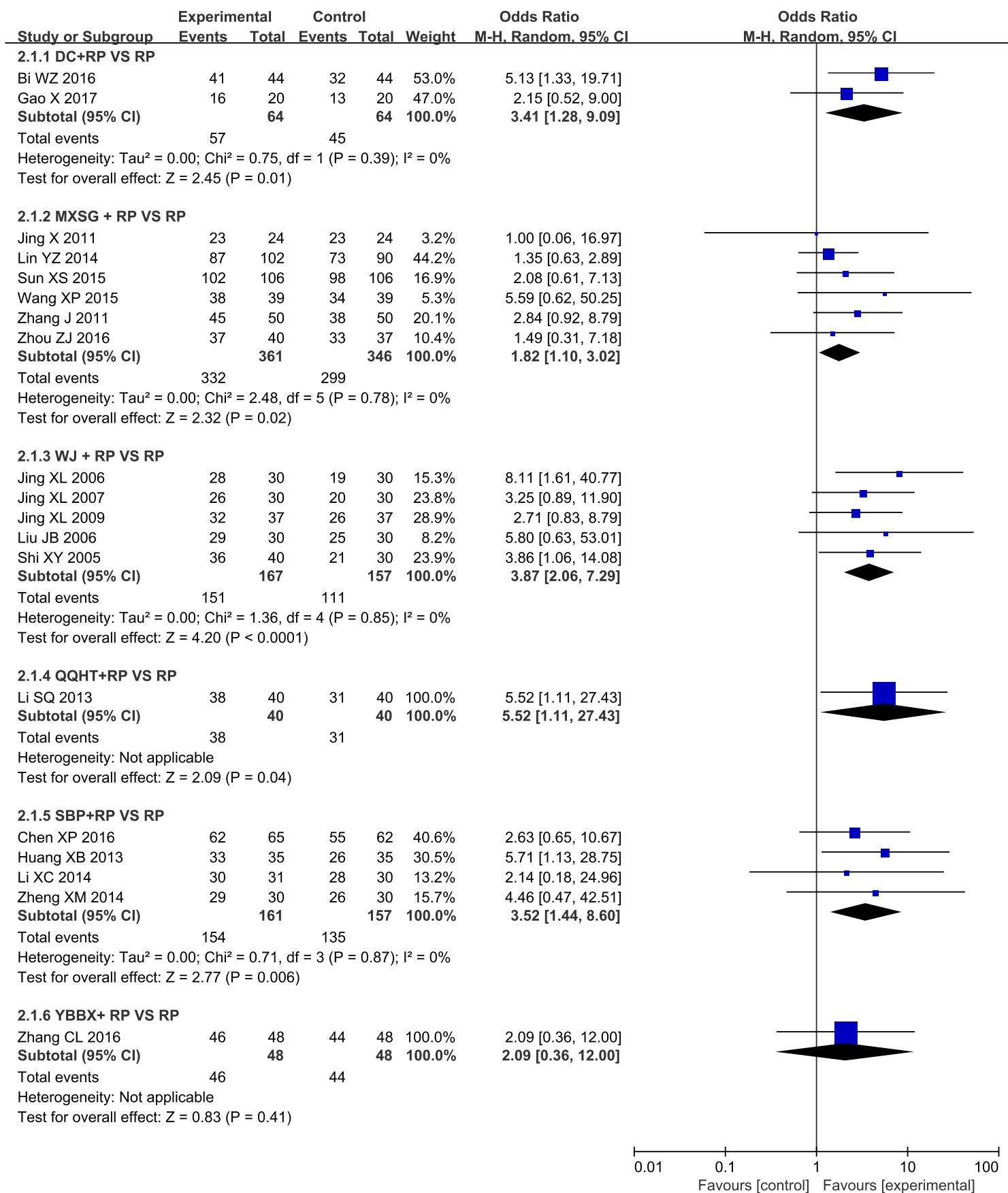

Supplement: Supplementary file 5 — Sensitivity analysis for effective rate. (PDF 1212 kb) [file 12906_2019_2633_MOESM5_ESM.pdf]

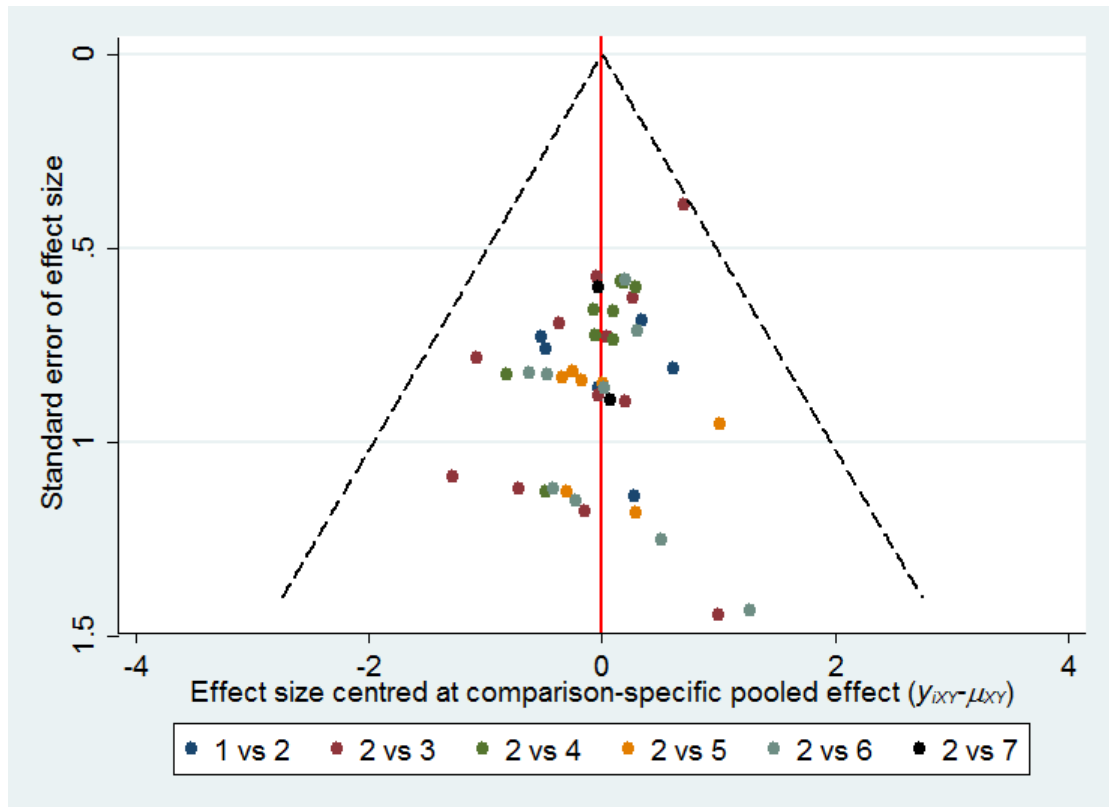

1=DC+RP, 2=RP, 3=MXSG+RP, 4=WJ+RP, 5=QQHT+RP, 6=SBP+RP,  
7=YBBX+RP

Supplement: Supplementary file 6 — Funnel plot of effective rate. (PDF 15 kb) [file 12906_2019_2633_MOESM6_ESM.pdf]
